# ^89^Zr-leukocyte labelling for cell trafficking: in vitro and preclinical investigations

**DOI:** 10.1186/s41181-023-00223-1

**Published:** 2023-11-06

**Authors:** Maryke Kahts, Hua Guo, Harikrishna Kommidi, Yanping Yang, Haluk Burcak Sayman, Beverley Summers, Richard Ting, Jan Rijn Zeevaart, Mike Sathekge, Omer Aras

**Affiliations:** 1 Pharmaceutical Sciences Department, School of Pharmacy, Sefako Makgatho Health Sciences University, Ga-Rankuwa, 0208 South Africa; 2 Department of Radiology, Molecular Imaging Innovations Institute (MI3), Weill Cornell Medicine, New York, NY 10065 USA; 3 State Key Laboratory of Molecular Oncology, National Cancer Center/National Clinical Research Center for Cancer/Cancer Hospital, Chinese Academy of Medical Sciences and Peking Union Medical College, Beijing, 100021 China; 4 Department of Nuclear Medicine, Cerrahpasa Medical Faculty, Istanbul University, 34303 Fatih, Istanbul Turkey; 5 Radiochemistry, The South African Nuclear Energy Corporation, Pelindaba, Hartebeespoort, 0240 South Africa; 6 Nuclear Medicine Research Infrastructure (NuMeRI), Department of Nuclear Medicine, Steve Biko Academic Hospital, University of Pretoria, Pretoria, South Africa; 7 DST/NWU, Preclinical Drug Development Platform, North West University, Potchefstroom, 2520 South Africa; 8 Department of Radiology, Memorial Sloan Kettering Cancer Center, New York, NY 10065 USA

**Keywords:** Cell trafficking, Infection imaging, Inflammation, PET, Zirconium-89

## Abstract

**Background:**

The non-invasive imaging of leukocyte trafficking to assess inflammatory areas and monitor immunotherapy is currently generating great interest. There is a need to develop more robust cell labelling and imaging approaches to track living cells. Positron emission tomography (PET), a highly sensitive molecular imaging technique, allows precise signals to be produced from radiolabelled moieties. Here, we developed a novel leukocyte labelling approach with the PET radioisotope zirconium-89 (^89^Zr, half-life of 78.4 h). Experiments were carried out using human leukocytes, freshly isolated from whole human blood.

**Results:**

The ^89^Zr-leukocyte labelling efficiency ranged from 46 to 87% after 30–60 min. Radioactivity concentrations of labelled cells were up to 0.28 MBq/1 million cells. Systemically administered ^89^Zr-labelled leukocytes produced high-contrast murine PET images at 1 h–5 days post injection. Murine biodistribution data showed that cells primarily distributed to the lung, liver, and spleen at 1 h post injection, and are then gradually trafficked to liver and spleen over 5 days. Histological analysis demonstrated that exogenously ^89^Zr-labelled human leukocytes were present in the lung, liver, and spleen at 1 h post injection. However, intravenously injected free [^89^Zr]Zr^4+^ ion showed retention only in the bone with no radioactivity in the lung at 5 days post injection, which implied good stability of radiolabelled leukocytes in vivo.

**Conclusions:**

Our study presents a stable and generic radiolabelling technique to track leukocytes with PET imaging and shows great potential for further applications in inflammatory cell and other types of cell trafficking studies.

**Supplementary Information:**

The online version contains supplementary material available at 10.1186/s41181-023-00223-1.

## Background

In vivo immune cell tracking by conventional methods can be complicated and invasive (Sato et al. 2015; Kurebayashi et al. 2021). As an alternative to these methods, imaging has emerged as an attractive non-invasive solution (Bansal et al. 2015; Kiraga et al. 2021), whereby the radiolabelling of cells alongside imaging can enable cell tracking throughout the whole body with high target-to-background ratios (Palestro 2023). Previously, indium-111 and technetium-99 m have been used with single photon emission computed tomography (SPECT) imaging for this purpose (Djekidel et al. 2011; Graute et al. 2010; Erba et al. 2012; Holcman et al. 2023). However, positron emission tomography (PET) imaging offers much higher sensitivity as well as higher spatial and temporal resolution than SPECT in tracking small numbers of cells administered to the body (Palestro 2023). This has motivated scientists in the search for a suitable PET radioisotope to label cells (Kiraga et al. 2021; Kusmirek et al. 2020; Fairclough et al. 2016; Pantin et al. 2015; Deri et al. 2013; McCracken et al. 2016). While fluorine-18 has been investigated for cell labelling, unfortunately, like many other PET radioisotopes, its short half-life (109.8 min) impeded its successful utility for long-term cell tracking (Sato et al. 2015; Palestro 2023; Fairclough et al. 2016; Petrik et al. 2016). By contrast, zirconium-89 (^89^Zr) is a long-lived, cyclotron-produced PET radioisotope with a half-life of 78.4 h. Thus, it is a superior isotope for cell tracking over several days to weeks (the latter allowing cell tracking over the critical early engraftment period of transplanted cells to the bone marrow) at a high sensitivity, resolution, and specificity (Bansal et al. 2015; Kiraga et al. 2021; Deri et al. 2013; Petrik et al. 2016; Lee et al. 2020). ^89^Zr can be obtained with high radionuclide purity and at high yields. ^89^Zr benefits from its relatively low mean positron emission energy of 395.5 keV, which translates into high image resolution (Fairclough et al. 2016; Deri et al. 2013; Sarcan et al. 2021).

In the context of monitoring and treating infection, which is a leading cause of death (Palestro 2023; Eggleston and Panizzi 2014), gallium-67 was one of the first SPECT-based agents used in infection imaging and is still being used today (Vorster et al. 2016). Currently, SPECT-based technetium-99 m (^99m^Tc) or indium-111 (^111^In) are also used to label leukocytes and are generally regarded as the “gold standard” in infection imaging, as they will localize at the sites of active infection through enhanced extravasation and diapedesis of leukocytes (Fairclough et al. 2016; Eggleston and Panizzi 2014; Takeuchi et al. 2016; Meibom et al. 2022). The radiolabelling of leukocytes with both [^111^In] oxine and technetium-99 m hexamethylpropyleneamine oxime ([^99m^Tc]Tc-HMPAO) are based on the formation of a lipophilic complex that can cross leukocyte cell membranes and become trapped in the cell. [^111^In] oxine dissociates within the cell, where indium-111 then binds to lactoferrin in the cytoplasm and the oxine component is released from the cells (Roca et al. 2010). The lipophilic [^99m^Tc]Tc-HMPAO complex is reduced to a hydrophilic complex after crossing the leukocyte cell membrane, which results in its entrapment within the cell (Vries et al. 2010). The most prominent disadvantages of the labelling approach for [^111^In] oxine- and [^99m^Tc]Tc-HMPAO-labelled leukocytes is possible radiation toxicity to intracellular components and gradual efflux of the radiolabel from the cell (Bansal et al. 2015).

Conventionally, infection imaging is performed at 1 h, 4 h, and 24 h after radiopharmaceutical administration, with the purpose of differentiating between inflammation, acute and chronic infections, but this time period is not always adequate to reliably detect infection (Bansal et al. 2015). While [^111^In] oxine (half-life of 67.2 h) is most commonly used to label cells as it offers a relatively long observation period, unfortunately, this agent has drawbacks, including a lower spatial resolution on SPECT and efflux due to the dissociation of the radiopharmaceutical from its chelator, which can occur in the specialized environments of sub-cellular organelles (Palestro 2023; Charoenphun et al. 2015). [^18^F]fluorodeoxyglucose ([^18^F]FDG) is used routinely in PET imaging of fever of unknown origin usually caused by infection (Takeuchi et al. 2016), but its relatively short half-life of 110 min restricts its utility in long-term cell tracking studies, and it also requires metabolically active cells (Palestro 2023).

This study focused on the labelling of leukocytes (white blood cells) with zirconium-89 using *p*-isothiocyanatobenzyl-desferrioxamine B (Df-Bz-NCS) to allow metal chelation and binding to biomolecules. The use of Df-Bz-NCS as bifunctional chelating agent to label various antibodies, proteins and stem cells with zirconium-89 is well documented (Moisio et al. 2022; Pandey et al. 2022; Zhang et al. 2022; Burvenich et al. 2021; Sobol et al. 2021; Bansal et al. 2020; Berg et al. 2020; Buckway et al. 2020; Cho et al. 2020; Lee et al. 2020). The isothiocyanate group of Df-Bz-NCS forms covalent thiourea bonds with primary amine groups on the cell surface (Perk et al. 2010), while there is strong chelation of ^89^Zr by desferrioxamine via three hydroxamate groups (Bansal et al. 2015). The covalent binding of the [^89^Zr]Zr-Df-Bz-NCS complex to the cell surface translates to greater stability of the label, when compared to conventional leukocyte labelling methods where the radiolabel has to cross the cell membrane and can gradually leak from the cell (Bansal et al. 2015). Another advantage of this labelling approach is the position of the radiolabel on the cell surface, rather than inside the cell as with conventional leukocyte labelling approaches, which avoids potential effects of the radiolabel on sensitive cell processes or components that might alter cell function or viability (Bansal et al. 2015). The leukocyte labelling method utilised in this study was based on the [^89^Zr]Zr-Df-Bz-NCS labelling approach described in literature [3; 25–35]. Our approach was to reduce the labelling time, by removing the incubation step for the formation of the [^89^Zr]Zr-Df-Bz-NCS complex. As mentioned previously, the use of Df-Bz-NCS as bifunctional chelating agent has been applied to label various antibodies, proteins and stem cells with ^89^Zr, but not yet for leukocyte labelling, as was performed in this study.

^89^Zr-leukocyte labelling showed high labelling efficiency and stability, and was able to track leukocytes in vitro as well as in vivo in an animal model. The results suggest that this effective and safe radiolabelling approach can be translated into clinical trials and used potentially for monitoring inflammatory diseases.

## Methods and materials

The current study aimed to successfully label leukocytes with ^89^Zr using *p*-isothiocyanatobenzyl-desferrioxamine B as bifunctional chelator, and to investigate the normal biodistribution of the ^89^Zr-labelled leukocytes in an animal model. The study was laboratory-based, experimental and analytical. The in vitro work was conducted at the Nuclear Medicine Department of the Steve Biko Academic Hospital and the preclinical investigations were performed at the Molecular Imaging Innovations Institute (MI3), in the Radiology Department of Weill Cornell Medicine.

### In vitro study

#### Leukocyte separation from whole blood

Peripheral blood samples were drawn from eight healthy volunteers and two patients with suspected and confirmed infection, respectively, into BD Vacutainer® citrate tubes (#: 369,714; Becton, Dickinson and Company, Franklin Lakes, NJ, USA) after ethical approval was obtained from the institutional review board at the Sefako Makgatho Health Sciences University (Ref no. SMUREC/P/21/2017) and informed consent was given. The 45 mL whole-blood sample from each participant was divided into two 50 mL-Falcon tubes (Greiner Bio-One, Kremsmünster, Austria) each containing 15 mL Ficoll blood separation medium (Ficoll-Paque™ PREMIUM; Cytiva Life Sciences™, Marlborough, MA, USA) and the sample was centrifuged at 800 g for 15 min to isolate the mononuclear layer (Heraeus™ Megafuge™ 8 centrifuge; Thermo Fisher Scientific, Waltham, MA, USA). The isolated mononuclear layers from both tubes were removed with a plastic Pasteur pipette, transferred to a clean 50 mL Falcon tube, and washed twice with 10 mL phosphate-buffered saline (PBS; pH = 7.4) (centrifuged at 250 g for 10 min each time). The washed cells were dislodged in 300 µL PBS for labelling.

In the study, we chose to use mononuclear cells because of their specific relevance to the research question. Mononuclear cells are a subset of white blood cells that include lymphocytes and monocytes and are primarily involved in immune responses. They play a key role in imaging infection and inflammation, as they are a crucial component of the body’s defense mechanisms (Kleiveland 2015). The choice of mononuclear cells was based on the objective of focusing on these immune cells to understand their role in infection and inflammation. It allowed us to narrow down the investigation to a specific cell population that has a prominent involvement in these processes. The isolation of mononuclear cells also offer a much faster and simpler method to obtain leukocytes for radiolabelling. The method described above for the isolation of mononuclear cells from whole blood takes approximately 40 min to complete, in comparison to 75 to 90 min for the conventional leukocyte separation technique via red blood cell sedimentation and centrifugation.

#### ^89^Zr-labelling

[^89^Zr] oxalic acid was imported by PerkinElmer South Africa (Pty) Ltd (Midrand, South Africa) from the Netherlands (BV Cyclotron VU, Amsterdam, The Netherlands). [^89^Zr] oxalic acid of various volumes (ranging from 7.4 µL to 34 µL) and activities (ranging from 4.74 MBq to 27.34 MBq) were used for labelling to investigate whether its volume and/or activity would affect its labelling efficiency. An equal volume of 2.0 M sodium carbonate (Na_2_CO_3_) (Sigma-Aldrich, St Louis, MO, USA) was added to each [^89^Zr]Zr^4+^ solution to neutralize ^89^Zr. Subsequently, 4 µL dimethyl sulfoxide (DMSO) (Life Technologies Corporation, Carlsbad, CA, USA) was gently mixed with 0.3 mg isothiocyanatobenzyl-desferrioxamine (Df-Bz-NCS) (Cas# 1,222,468–90-7; Macrocyclics, Inc., Plano, Texas, USA) and the resulting mixture was centrifuged at 1000 rpm for 1 min to form a pellet. The supernatant of the DMSO-Df-Bz-NCS mixture was removed without disturbing the pellet and added to an Eppendorf tube (Greiner Bio-One, Kremsmünster, Austria) containing neutralized [^89^Zr]Zr^4+^, followed by the addition of 275 µL PBS. The pH of the solution was adjusted with 2.0 M Na_2_CO_3_ and/or 1.0 M hydrochloric acid (HCl) (Sigma-Aldrich, St Louis, MO, USA) to obtain a final pH of 7. Then, washed leukocytes were added to the neutral-pH [^89^Zr]Zr-Df-Bz-NCS solution and the resulting mixture was incubated for 30 to 60 min at 37 °C. After incubation, the resulting ^89^Zr-labelled leukocytes were washed twice with 5 mL PBS and resuspended in the desired volume of PBS.

#### Molar activity, labelling efficiency and yield determination

The apparent molar activity of the [^89^Zr]Zr-Df-Bz-NCS complex before leukocyte labelling was determined, by taking into account the number of moles of the bifunctional chelate and the activity of ^89^Zr used for each labelling attempt (Coenen et al. 2019). ^89^Zr activities in the supernatant and the cells were measured using a CURIEMENTOR® 4 dose calibrator (PTW, Freiburg, Germany), and labelling efficiencies were calculated, expressed as the percentage of the ratio between the radioactivity associated with the leukocytes and the total radioactivity (Gawne et al. 2022). The accepted formula to calculate the labelling efficiency of cells is presented below:

$$Labelling \,Efficiency \left(\%\right)= \frac{Activity \,in \,the \,cells \,(MBq)}{Activity \,in \,the \,combined \,supernatants \,(MBq)+Activity \,in \,the \,cells \,(MBq) } \times 100$$

The labelling yield was determined by measuring the amount of radioactivity incorporated in the final suspension of leukocytes at the end of the [^89^Zr]Zr-Df-Bz-NCS-leukocyte labelling and expressing it as a percentage of the initial ^89^Zr activity used for each labelling attempt (Coenen et al. 2019).

To confirm cell-associated ^89^Zr, the labelled cells underwent a freeze–thaw cycle and lysed cells were centrifuged. The radioactivity in the supernatant and pellet were then gamma counted to determine the percentage of radioactivity that was trapped in the pellet. From this experiment, we found that about 80–85% of the radioactivity was recovered in the pellet, providing evidence for cell-associated ^89^Zr labelling (data not shown).

Other quality control tests on the [^89^Zr]Zr-Df-Bz-NCS complex and the ^89^Zr-labelled leukocytes were not performed, due to constraints in the availability of the relevant equipment in the radiopharmacy where the labelling was conducted. These quality control parameters include chelation efficiency, cell viability after labelling and composition of the mononuclear cells before labelling. However, many of these parameters (i.e. cell viability and function, chelation efficiency and stability of the labelled cells) were indirectly assessed via the initial biodistribution and eventual migration of the labelled cells in the animal model chosen for this study.

### Preclinical study

#### PET imaging

Female Balb/c mice (5 weeks old, 20–25 g) were purchased from Jackson Laboratories (Bar Harbor, ME, USA). The choice of the Balb/c mouse model for tracking human ^89^Zr-labelled leukocytes in live animals was based on several considerations that are fundamental to the study design. Here are some key points to clarify the rationale behind this choice:

Immunodeficiency: Balb/c mice are known for their specific immune system characteristics that make them suitable for studies involving human cells. In this case, the choice of Balb/c mice allowed for the tracking of human ^89^Zr-labelled leukocytes without triggering significant immunological responses, as these mice have a less robust immune system compared to other strains.

Compatibility: Human ^89^Zr-labelled leukocytes can be more effectively engrafted into the Balb/c model due to the compatibility of the mouse strain with human cells, which facilitates the tracking and analysis of these cells in vivo.

Consistency: Balb/c mice are widely used in immunology and transplantation studies, making them a well-established model for these types of investigations. This consistency in the model choice helps ensure reliable and reproducible results.

Translational Relevance: The choice of Balb/c mice for this study may have translational relevance, as it could potentially provide insights into how human ^89^Zr-labelled leukocytes might behave in a setting that somewhat mimics human physiology.

All animal procedures were approved by the institutional animal care and use committee at Weill Cornell Medicine in New York, NY, USA (No. 2014–0030), and were consistent with the recommendations of the American Veterinary Medical Association and the United States National Institutes of Health Guide for the Care and Use of Laboratory Animals.

Mice (n = 11) were anesthetized with 1.5%–2.5% isoflurane and injected via the tail vein with 0.22 MBq (100 µL) of ^89^Zr-labelled leukocytes isolated from a healthy volunteer (approximately 0.28 MBq/1 × 10^6^ cells in 2 mL suspension). The anesthetized mice were placed on a tray for imaging with an Inveon® PET/CT system (Siemens AG, Munich, Germany). A 10-min CT/ 30-min PET scan was acquired at 1 h, 24 h, and 5 days post injection. PET/CT data, stored as Digital Imaging and Communications in Medicine (DICOM) images, were processed with AMIDE v1.0.4 software. [^89^Zr]Zr^4+^ ion that was introduced intravenously (n = 2) served as a control. Following the completion of imaging, the mice (n = 3, each time point) were sacrificed by cervical dislocation. Brain, blood, heart, lung, liver, spleen, stomach, intestine, muscle, bone (femur) and fat tissues were harvested, weighed, and transferred to test tubes for scintigraphy using a Wallac Wizard 3.0 gamma counter (PerkinElmer, Finland). Following scintillation, tissues were fixed in 4% of PFA/PBS for further histological analysis.

#### Histology

The fixed harvested tissues that were obtained from the sacrificed mice were embedded in paraffin and 7-µm slices were cut using a microtome (RM 2155; Leica, Germany). Slices were then immunohistochemically stained with anti-human CD45 antibody (#M0701; Dako, Carpinteria, CA, USA) and incubated for 60 min with biotinylated horse anti-mouse IgG (#MKB-2225B; Vector Laboratories, Newark, CA, USA). Immunohistochemical staining was performed at the Molecular Cytology Core Facility at Memorial Sloan Kettering Cancer Center in New York, NY, USA, using a Discovery XT processor (Ventana Medical Systems, Oro Valley, AZ, USA). Detection was performed using a DAB detection kit (Ventana Medical Systems) according to manufacturer instructions. Slides were counterstained with hematoxylin and coverslipped with Permount (Thermo Fisher Scientific).

All instruments and equipment used in the study were calibrated to ensure accurate measurements and reliable results.

## Results

### Apparent molar activity, labelling yield and in vitro labelling efficiency

The molecular weight of the Df-Bz-NCS bifunctional chelating agent (C_33_H_52_N_8_O_8_S_2_) was 752.9 g/mol, as specified on the Product Safety Data Sheet supplied by Macrocyclics. The average apparent molar activity of the [^89^Zr]Zr-Df-Bz-NCS complex used for leukocyte labelling was calculated as 34.95 ± 18.28 MBq/µmol (see Table 1). Overall, the labelling efficiency ranged from 46 to 87% (average = 57 ± 12.46%), independent of ^89^Zr activity and volume (see Table 1). The number of leukocytes in the blood sample was suspected to be the determining factor for labelling efficiency, as the highest labelling efficiency (87%) was obtained in the blood of the patient with confirmed infection (diabetic foot). Similar labelling efficiencies were achieved in published studies on [^89^Zr]Zr-Df-Bz-NCS-labelled cells. Bansal et al. achieved labelling efficiencies of 30 to 50% for mouse-derived melanoma cells, dendritic cells and human mesenchymal stem cells labelled with [^89^Zr]Zr-Df-Nz-NCS (Bansal et al. 2015); Lee et al. reported high labelling efficiencies of 70% to 79% for [^89^Zr]Zr-DFO-labelled CAR T-cells (Lee et al. 2020); and Bansal et al. reported labelling efficiencies of 30% to 40% for [^89^Zr]Zr-DBN-labelled cardiopoietic stem cells (Bansal et al. 2020).

**Table 1:** Apparent molar activity of the [^89^Zr]Zr-Df-Bz-NCS complex (MBq/µmol), labelling efficiency (%) and yield (%) of ^89^Zr-labelled leukocytes isolated from two patients and eight healthy volunteers

Patient/healthy volunteer no	^89^Zr activity (MBq)	Volume of ^89^Zr oxalic acid (µL)	Apparent molar activity of the [^89^Zr]Zr-Df-Bz-NCS complex (MBq/µmol)	Incubation period at 37 °C (min)	Labelling yield (%)	Labelling efficiency (%)
1	7.92	7.4	19.88	45	48	46
2	7.96	9.2	19.98	30	61	59
3	15.91	22.4	39.93	30	79	87
4	21.72	34.0	54.51	30	45	53
5	17.87	10.9	44.85	40	16	52
6	27.34	13.5	68.61	45	38	50
7	19.98	10.0	50.14	60	40	47
8	6.33	12.0	15.89	60	54	74
9	9.47	20.0	23.77	60	37	52
10	4.74	20.0	11.90	60	42	52
Average	13.92	15.9	34.95	46	46	57
SD	7.28	7.75	18.28	12.61	15.75	12.46

The average labelling yield was 46 ± 15.75%, with a range of 16% to 79% (see Table 1). The major contributors to loss of ^89^Zr activity during the labelling process, which may affect the labelling yield, were the known “stickiness” of ^89^Zr and its resultant adherence to surfaces of vials, etc. during labelling (Massicano et al. 2020), as well as pH adjustment of the [^89^Zr]Zr-Df-Bz-NCS complex. After chelation, the pH of the [^89^Zr]Zr-Df-Bz-NCS complex must be neutralised to a pH of 7 for cell labelling. The pH adjustment step required small samples to be taken from the [^89^Zr]Zr-Df-Bz-NCS solution and testing the pH of these samples until the required pH of 7 was achieved. There were obvious losses of ^89^Zr activity with each sample taken for pH testing. Radioactivity concentration was determined to be approximately 0.28 MBq/1 million cells before intravenous injection into the mice.

### Small-animal PET imaging

**Fig. 1:**
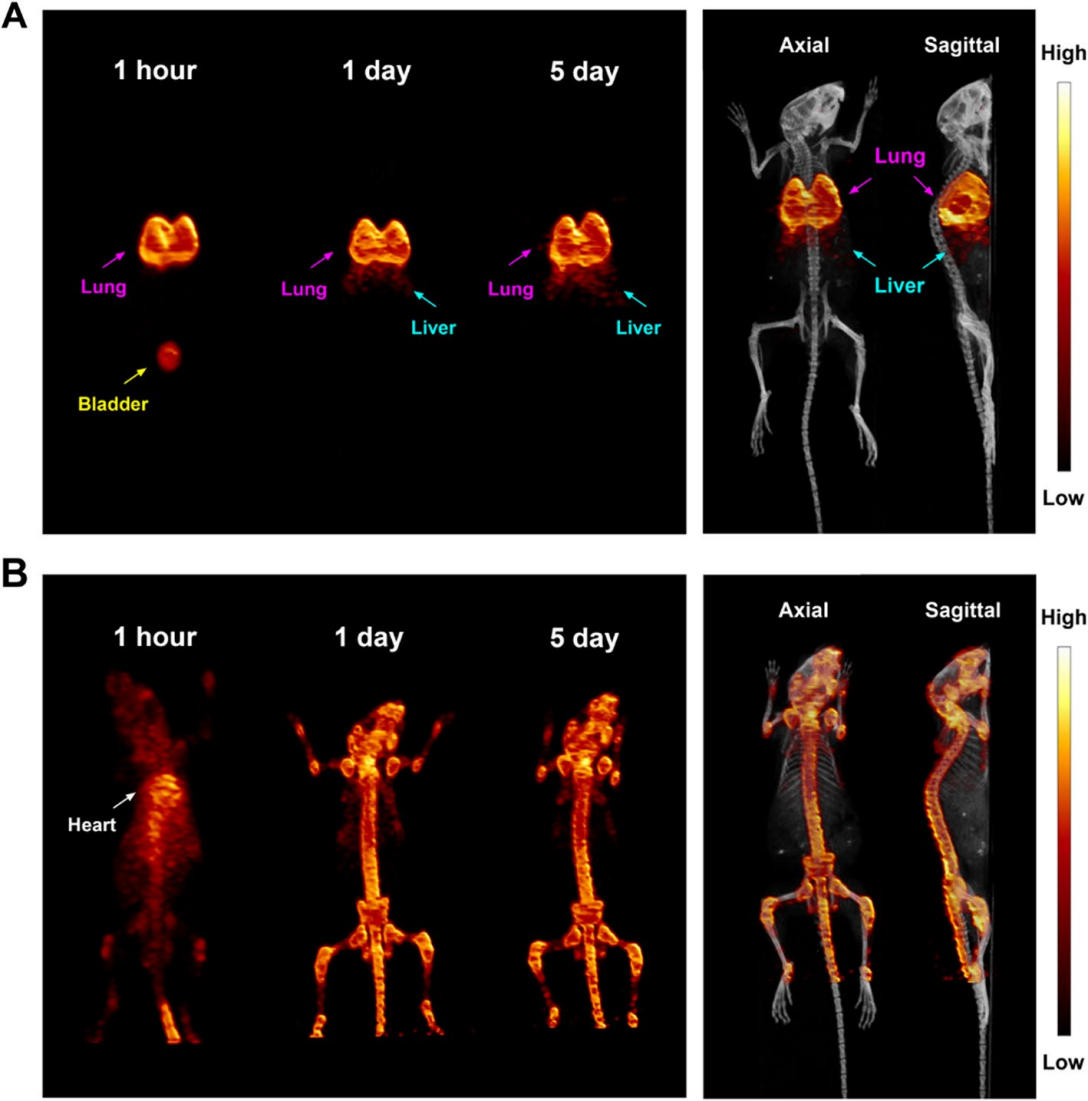
Maximum intensity projection (MIP) PET scan showing that intravenously introduced ^89^Zr-labelled leukocytes accumulated in the lung at 1 h, 1 day, and 5 days post injection. 5-week-old female Balb/c mice were intravenously injected with 0.22 MBq ^89^Zr-labelled leukocytes (**A**) or free [^89^Zr]Zr^4+^ ion (**B**, control) and 30 min PET scans were performed at 1 h, 1 day, and 5 days post injection. Right panels show representative PET/CT images at 5 days post injection

To track the human ^89^Zr-labelled leukocytes in live, healthy Balb/c mice, a Siemens Inveon small-animal PET scanner was used to visualize the labelled leukocytes in vivo after the mice each received a 0.22 MBq intravenous dose of ^89^Zr-labelled leukocytes. Initial distribution to the lungs was observed at 1 h post injection. Lung activity remained high for up to 5 days. Distribution in other tissues and organs was low (Fig. 1A, Additional file 1: video 1). A slight trend of ^89^Zr-leukocytes migrating to the liver over time was observed. As a control, to show that the [^89^Zr]Zr^4+^ ion does not dissociate from leukocytes, intravenous free [^89^Zr] oxylate ([^89^Zr]Zr^4+^ ion) was injected to show initial high activity in the heart and blood pool at 1 h, followed by gradual accumulation in the bone from day 1 to day 5, but not in the lung (Fig. 1B, Additional file 1: video 1). As no radioactivity was observed in the bone after intravenously delivered ^89^Zr-leukocytes, it can be concluded that our approach for radiolabelling of leukocytes has good stability in vivo and no free [^89^Zr]Zr^4+^ ion would detach from the leukocytes after administration.

### Tissue biodistribution

**Fig. 2:**
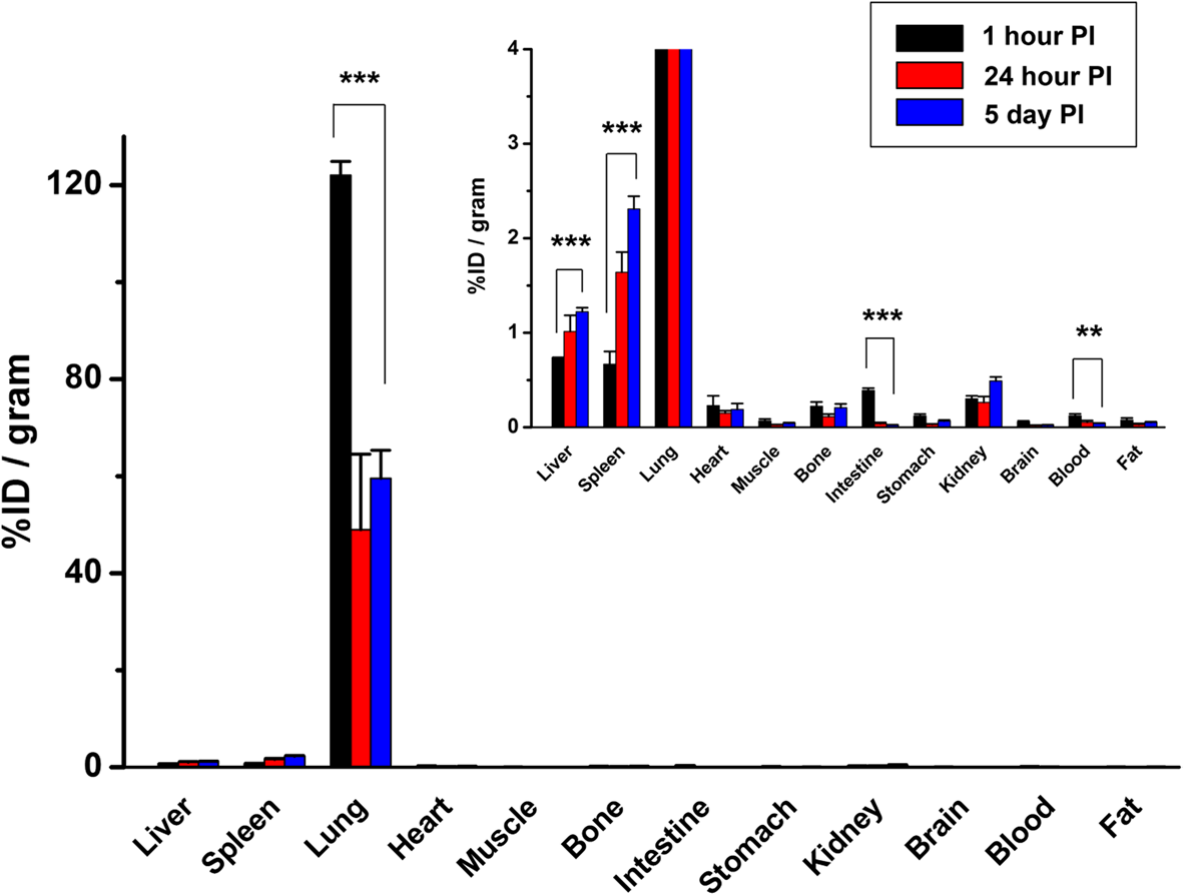
Scintillated biodistribution of the main organs at 1 h (n = 3), 24 h (n = 3), and 5 day (n = 4) post intravenous injection of 0.22 MBq ^89^Zr-labelled leukocytes. Data are shown as mean ± standard error of the mean. ***: *P* < 0.001, **: *P* < 0.01. PI, post injection

**Fig. 3:**
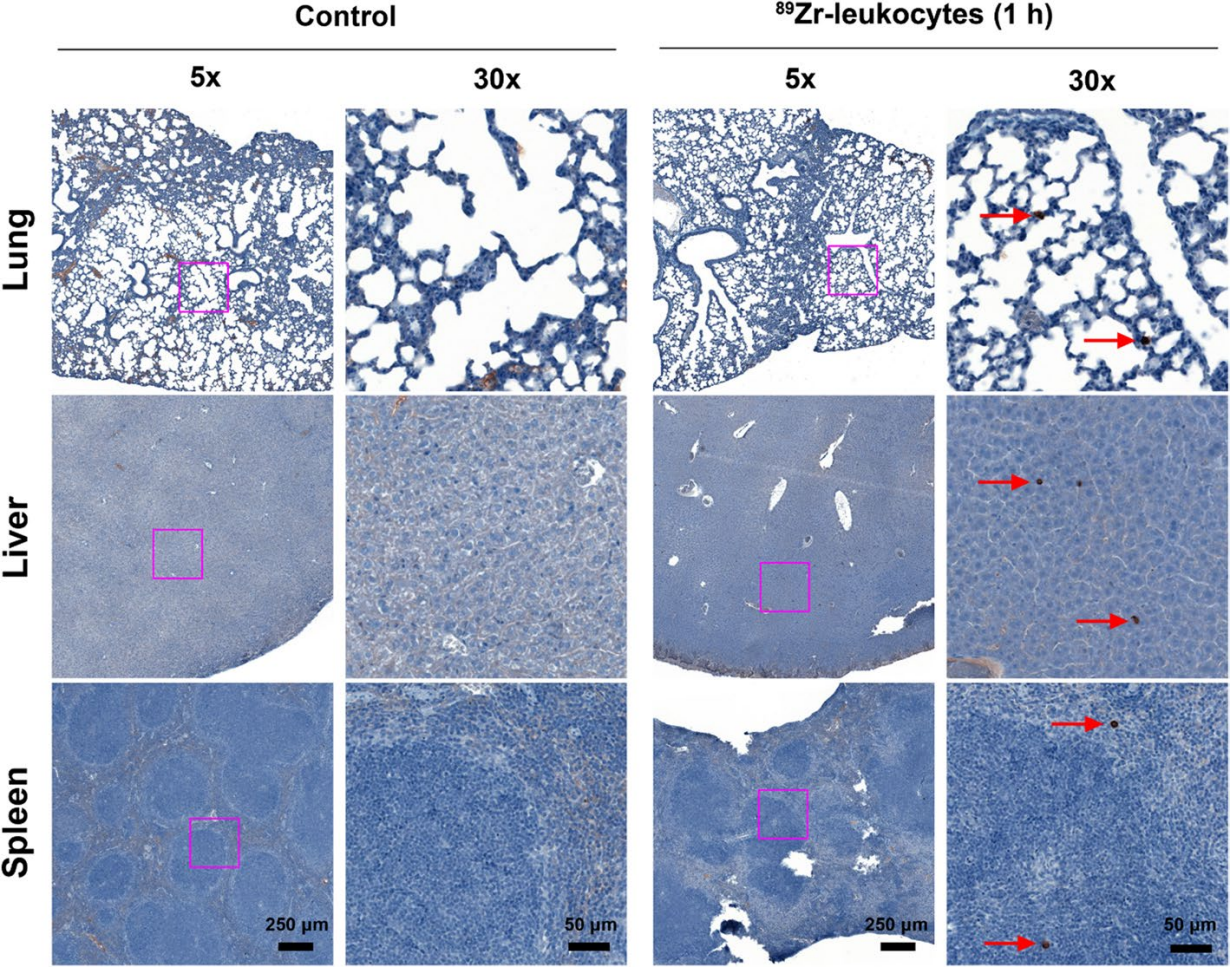
Representative immunohistochemical staining showing that exogenously ^89^Zr-labelled human leukocytes are present in lung, liver, and spleen of the mice following 1 h post-intravenous administration of ^89^Zr-leukocytes. Mice without treatment served as controls. The magenta box in the 5 × column was magnified in 30 × column. Red arrows indicate human leukocytes

In vivo tissue biodistribution was evaluated at 1 h, 24 h, and 5 days post injection of ^89^Zr-leukocytes (Fig. 2). Tissue uptake was highest in the lungs, peaking at 1 h (122.0 ± 2.9%ID/g) and falling to 49.0 ± 15.5%ID/g by day 1, but the radioactivity remained high at 59.6 ± 5.7%ID/g at day 5, which was consistent with PET data. Liver and spleen uptake showed a marked increase from 1 h to day 5 (*P* values < 0.0005), which demonstrated that ^89^Zr-leukocytes gradually trafficked to the liver and lung. Reduced radioactivity was shown in the blood, stomach, and intestines over time. No statistical difference of radioactivity was found in the kidney, brain, heart, muscle, bone, or fat throughout the duration of the study. Quantities of intravenously introduced [^89^Zr]Zr^4+^ ion were eliminated from the lung quickly and gradually accumulated in the bone over time (Fig. 1B). These data confirm that ^89^Zr distribution to the lung, liver and spleen is representative of ^89^Zr-leukocyte trafficking. As PET imaging and biodistribution data demonstrated that the leukocytes accumulated in the lung, liver, and spleen, we further collected those organs for human leukocyte immunohistochemical staining. From the representative tissue sample images shown in Fig. 3, exogenous ^89^Zr-labelled human leukocytes were found in the lung, liver, and spleen of the mice following 1 h post-intravenous administration (pointed by red arrows). These results clearly indicate the possibility of our radiolabelling technique to track leukocytes by PET imaging.

If damaged cells were indeed trapped in the lungs, one might expect to observe some free ^89^Zr elsewhere in the body, indicating the release of the radiolabel from these cells. Furthermore, damaged cells are typically processed and cleared by the body's immune system, including the liver and spleen, which should prevent the long-term accumulation of damaged cells in the lungs. A prolonged and elevated uptake of human ^89^Zr-labelled white blood cells (WBC) in the lungs of mice could be attributed to several factors, and further investigation is required to understand the underlying mechanisms. Here are some potential explanations:

Species Mismatch: One possibility is that there may be species-specific interactions or differences in the behaviour of human WBCs in a mouse model. Human cells may not interact or clear in the same way that mouse cells do in their native host.

Lung-Specific Homing: White blood cells are involved in immune responses and can migrate to sites of infection or inflammation. It is possible that in this experimental model, human WBCs have a specific affinity for lung tissue. This could be due to the presence of lung-specific antigens or inflammation markers that attract these cells.

Immunological Factors: The immune system plays a critical role in clearing foreign cells. In this case, the immune response might not be efficiently recognizing or clearing the human WBCs from the lungs, leading to their prolonged retention.

## Discussion

The ideal PET tracer for labelling cells should have good retention of the radiotracer within the cells while preserving cell viability and function. Most importantly, the radiotracer should have a long-lived physical half-life (Mohammadpour-Ghazi et al. 2023). Zirconium-89 (t_½_ = 78 h), a positron emitting radionuclide of relatively low energy, is produced via the proton bombardment of naturally abundant yttrium-89 using a biomedical cyclotron (Sarcan et al. 2021; Mohammadpour-Ghazi et al. 2023; Gordon and Vivian 1984) and its production is characterized by high purity and high specific activity (Sarcan et al. 2021; Holland et al. 2009; Ikotun and Lapi 2011). In the current study, we described a method for efficient and rapid labelling of leukocytes with a PET-emitting, long-lived radioisotope, i.e., ^89^Zr. ^89^Zr-labelling was mediated by the Df-Bz-NCS bifunctional chelate, which binds to primary amines of cell surface protein, therefore indicating that this labelling method can be adapted to label a wide variety of cells. Cell labelling did not affect the viability or function of leukocytes. These results reveal that leukocytes can be labelled in vitro with zirconium-89. Labelling was optimized when the cells were incubated for a minimum of 30 min with the [^89^Zr]Zr-Df-Bz-NCS complex at 37 °C under neutral pH conditions. Moreover, upon administration of the ^89^Zr-labelled leukocytes to healthy Balb/c mice, initial distribution to the lungs was observed at 1 h post injection, which remained high for up to 5 days, with no accumulation in the bone. Free [^89^Zr] oxylate ([^89^Zr]Zr^4+^ ion) was injected intravenously as a control. Gradual accumulation in the bone was observed with no activity in the lungs. The controls indicated that the ^89^Zr-labelled leukocytes do not dissociate once administered and thus proved high in vivo stability.

In vivo cell tracking with the use of nuclear medicine imaging techniques has been an important diagnostic tool since it was developed more than 40 years ago with the introduction of labelled autologous cells (Lee et al. 2020; Gordon and Vivian 1984; McAfee and Thakur 1976; Rovekamp et al. 1981; Welling et al. 2019). Although few PET radioisotopes have been utilized, other methods to label gamma emitters have been widely used. The most widely employed methods are [^111^In] oxyquinoline (oxine) and [^99m^Tc]Tc-HMPAO. McAfee and Thakur (McAfee and Thakur 1976) first described the incubation with [^111^In] oxine as an efficient method for in vitro labelling of cells including granulocytes and platelets. Rannie et al. compared several unconjugated radiotracers (^99m^Tc, ^51^Cr, and [^111^In] oxine) for labelling freshly harvested lymphocytes in early clinical cell tracking studies and showed that ^111^In-labelled oxine was clearly the most promising for studying lymphocyte migration in patients (Rannie et al. 1977). This is due to the retention of [^111^In] oxine in vivo and in vitro within lymphocytes and the migration of the labelled cells into lymphoid tissues. These early studies made the labelling of human leukocytes with indium-111 possible to track these cells with the use of gamma cameras. ^111^In-labelling demonstrated both high sensitivity and specificity and its longer half-life (t_1/2_∼2.8 days) would be suitable to answer the question of the “fate” of cells for in vivo application, but the non-ideal physical characteristics of indium-111 made imaging suboptimal (Krishnaraju et al. 2021). The detection sensitivity of [^111^In] oxine-labelled cells in SPECT imaging was reported to be approximately 1000 (Lappalainen et al. 2008). As ^99m^Tc has a shorter half-life and lower energy than ^111^In, it has more optimal physical characteristics for imaging (Kelbaek 1986). In addition, ^99m^Tc has the advantages of having a lower radiation and cost, being widely available and being easy to handle.

PET imaging has a higher sensitivity and spatial resolution than SPECT imaging and permits more accurate quantification of cell numbers for PET-based cell tracking (Krishnaraju et al. 2021). Perhaps the most commonly used PET agent for cell tracking is [^18^F]FDG, but it has a relatively short half-life of 110 min and requires metabolically active cells. The advantages of labelling cells with [^18^F]FDG compared to other radiotracers are immediate clinical availability, relatively low radiation exposure, quicker results, availability of dynamic acquisitions and quantitative analysis, and high sensitivity and higher resolution when used in conjunction with PET imaging, resulting in precise determination of cell accumulation (Meier et al. 2008). It has been shown that the mean labelling efficiencies of [^111^In] oxine and [^18^F]FDG were higher than that of [^99m^Tc]Tc-HMPAO (Botti et al. 1997). These three radionuclides have been shown to induce no significant alteration in cell viability or immunophenotype, but both [^111^In] oxine and [^18^F]FDG caused a loss of cytotoxic activity of lymphocytes against ovarian carcinoma cells (Kelbaek 1986). Other studies with hematopoietic progenitor cells and bone marrow-derived mesenchymal stem cells demonstrated that at high concentrations, indium-111 has an inhibitory effect on these cells (Bindslev et al. 2006; Yoon et al. 2010). Copper-64 (t_½_ = 12.7 h) has a longer half-life but suffers from a relatively low yield of positrons, leading to high dose exposures of labelled cells. Given its longer half-life, ^64^Cu is passively delivered into cells by the lipophilic redox-active carrier molecule, pyruvaldehyde-bis [^4^N] methylthiosemicarbazone) (PTSM) (Adonai et al. 2002), and [^64^Cu]Cu-PTSM has been used to label tumour cells for non-invasive PET imaging studies of cell trafficking in mice (Adonai et al. 2002; Huang et al. 2008). The radiolabelling of [^64^Cu]Cu-PTSM on rhesus monkey CD34 + hematopoietic and mesenchymal stem cells proved to be safe with optimized concentrations of 7.4 GBq and 3.7 GBq/mL, respectively. However, higher doses (greater than 7.4 GBq/mL) resulted in growth delays and possible DNA damage, and at 14.8 GBq/mL, there was significant declining of osteogenic differentiation (Huang et al. 2008).

While various chelators have been exploited with ^89^Zr in the search for a stable radiometal chelate, the most success has been reported with desferrioxamine B (DFO), a hexadentate, bifunctional siderophore with three hydroxamate groups for metal chelating and a primary amine tail for binding to a biomolecule (Perk et al. 2010; Chomet et al. 2021). It has been reported that DFO binds rapidly and efficiently in a 1:1 ratio of metal to chelate and offers high stability with negligible dissociation (Bansal et al. 2015; Deri et al. 2013; Lee et al. 2020; Perk et al. 2010). Since free ^89^Zr, in its osteophilic [^89^Zr]Zr^4+^ cation form, is a bone-seeking agent by nature, the stability of the chelate complex is apparent when significantly less bone uptake is observed upon imaging. A derivative of DFO has been developed in recent years to further simplify labelling procedures, i.e., *p*-isothiocyanatobenzyl-bearing DFO (Df-Bz-NCS) (Kiraga et al. 2021; Fairclough et al. 2016; Chomet et al. 2021). [^89^Zr]Zr-Df-Bz-NCS binds to primary amines of cell surface protein expressed by all cells and can thus be considered a translational method to label a wide variety of cells. ^89^Zr is chelated by Df-Bz-NCS with three hydroxamate groups and there is inherent biostability of the thiourea bond that conjugates the NCS group in the radiopharmaceutical to primary amines of the cell surface protein. Wang and colleagues utilized a similar approach to radiolabel erythrocytes with ^18^F for PET imaging of intracranial haemorrhage (Wang et al. 2017). The labelling process involved the covalent binding of [^18^F]F^−^ ions to superficial amines on the outer cell membranes of the erythrocytes, which remained stable in vivo.

As the radiolabelling method with Df-Bz-NCS is more effective and safer than the [^89^Zr] oxine approach (as there is no efflux of the agent from the cells or intracellular processing of the agent), we used this method in our study. In this method, the majority of ^89^Zr does not enter into the cells but remains bound to the extracellular surface where pH and oxidative state are physiologically normal. Thus, we have developed a protocol for cell labelling with ^89^Zr, which results in optimal label retention, visualization on clinical PET imaging, and insignificant cytotoxicity. It should be feasible to translate [^89^Zr]Zr-Df-Bz-NCS labelling of human cells into clinical trials. The use of a radionuclide with a long half-life could be justified by the low amount of radioactivity that is needed to obtain satisfactory imaging results.

### Limitations

The chelation efficiency (radiochemical purity) and in vitro stability of the [^89^Zr]Zr-Df-Bz-NCS complex were not investigated directly in this study. The study limitations also included determination of the composition of the mononuclear cells isolated for labelling and assessment of the in vitro stability, viability and function of the ^89^Zr-labelled leukocytes. These parameters were indirectly assessed via the biodistribution and migration of the labelled cells during the preclinical investigations. There is need for further studies to assess these parameters directly.

## Conclusion

A robust method to radiolabel human leukocyte with ^89^Zr via the bifunctional chelate Df-Bz-NCS was investigated in this study. The long half-life of ^89^Zr offers the opportunity for prolonged cell migration imaging in vivo with high resolution, sensitivity, and quantification, as obtained using PET imaging. The observed biodistribution of ^89^Zr-labelled leukocytes is consistent with the results of other studies, i.e., initial uptake in the lungs, followed by the migration of cells to the liver and spleen. No uptake in the bone was observed, which is a testament to the stability of the ^89^Zr-agent and therefore the absence of free [^89^Zr]Zr^4+^. The results of this study present a stable and generic radiolabelling technique to track leukocytes in infection imaging using PET imaging and shows great potential for further applications in other types of cell trafficking studies, e.g., stem cells. A study involving PET imaging with ^89^Zr-labelled leukocytes in patients with infection is in process, following the labelling approach described in this paper. This study will provide a better understanding of the effect of the ^89^Zr label on the viability and function of leukocytes in active infections.

### Supplementary Information

**Additional file 1**. PET/CT images showing that intravenously introduced ^89^Zr-labelled leukocytes accumulated in the lung at 1 day and 5 days post injection, with slight migration to the liver over time. Free zirconium-89 accumulated in bone with no lung uptake. 5-week-old female Balb/c mice were intravenously injected with 0.22 MBq ^89^Zr-labelled leukocytes or free [^89^Zr]Zr^4+^ ion (control) and 30 min PET scans were performed at 1 day and 5 days post injection.

## Data Availability

The datasets used and/or analysed during the current study are available from the corresponding author on reasonable request.

## Abbreviations

^18^F: Fluorine-18
[^18^F]FDG: ^18^Fluorine-fluorodeoxyglucose
^51^Cr: Chromium-51
^64^Cu: Copper-64
^89^Zr: Zirconium-89
^99m^Tc: Technetium-99m
^111^In: Indium-111
DAB: 3,3*N*-Diaminobenzidine tertrahydrochloride
Df-Bz-NCS: *p*-Isothiocyanatobenzyl-desferrioxamine B
DFO: Desferrioxamine
DICOM: Digital imaging and communications in medicine
DMSO: Dimethyl sulfoxide
HCl: Hydrochloric acid
HMPAO: Hexamethylpropyleneamine oxime
IgG: Immunoglobulin G
keV: Kilo-electron-volt
MBq: Megabecquerel
MIP: Maximum intensity projection
Na_2_CO_3_: Sodium carbonate
PBS: Phosphate-buffered saline
PET: Positron emission tomography
PET/CT: Positron emission tomography/computed tomography
PFA: Paraformaldehyde
PTSM: Pyruvaldehyde-bis [^4^N] methylthiosemicarbazone
SPECT: Single photon emission computed tomography
